# Open-Source Data Collection and Data Sets for Activity Recognition in Smart Homes

**DOI:** 10.3390/s20030879

**Published:** 2020-02-06

**Authors:** Uwe Köckemann, Marjan Alirezaie, Jennifer Renoux, Nicolas Tsiftes, Mobyen Uddin Ahmed, Daniel Morberg, Maria Lindén, Amy Loutfi

**Affiliations:** 1 Centre for Applied Autonomous Sensor Systems (AASS), Örebro University, 70182 Örebro, Sweden; marjan.alirezaie@oru.se (M.A.); jennifer.renoux@oru.se (J.R.); amy.loutfi@oru.se (A.L.); 2 RISE SICS, RISE Research Institutes of Sweden, 16440 Stockholm, Sweden; nicolas.tsiftes@ri.se; 3 School of Innovation Design and Engineering (IDT), Mälardalen University, 72220 Västerås, Sweden; mobyen.ahmed@mdh.se (M.U.A.); daniel.morberg@mdh.se (D.M.); maria.linden@mdh.se (M.L.)

**Keywords:** smart home data sets, data collection software, prototype installation

## Abstract

As research in smart homes and activity recognition is increasing, it is of ever increasing importance to have benchmarks systems and data upon which researchers can compare methods. While synthetic data can be useful for certain method developments, real data sets that are open and shared are equally as important. This paper presents the E-care@home system, its installation in a real home setting, and a series of data sets that were collected using the E-care@home system. Our first contribution, the E-care@home system, is a collection of software modules for data collection, labeling, and various reasoning tasks such as activity recognition, person counting, and configuration planning. It supports a heterogeneous set of sensors that can be extended easily and connects collected sensor data to higher-level Artificial Intelligence (AI) reasoning modules. Our second contribution is a series of open data sets which can be used to recognize activities of daily living. In addition to these data sets, we describe the technical infrastructure that we have developed to collect the data and the physical environment. Each data set is annotated with ground-truth information, making it relevant for researchers interested in benchmarking different algorithms for activity recognition.

## 1. Introduction

Ambient Assisted Living (AAL) applications are of increasing importance in ageing societies, as they help enable elderly and persons with special needs to live independently at home. Over the past decade, many technological advancements have been made in sensor networks, personal assistants, domestic robotics, and the Internet of Things. As a result, several relevant research projects have achieved proof-of-concept systems. An important concept in these systems is the ability to use sensors—either distributed in the environment or integrated on robots—to detect and recognize human behaviour. These include detecting activities of daily living, detecting anomalies (such as nighttime wakings), and detecting emergencies (such as falls).

This paper is the cumulative result of a five-year project called E-care@home, in which the goal was to develop methods and techniques for activity recognition in homes of elderly. The project provided a set of tools and software that are publicly available to be used by other researchers in the area.

The two contributions of this paper are as follows: first, we provide a modular and extendable architecture for an Internet of Things system (that we refer by the E-care@home system) focused on *semantic interoperability*. This contribution also contains a “cookbook” describing the technical steps to perform in order to setup an instantiation of the E-care@home system. Second, we provide 3 different data sets collected using the E-care@home system and targeted towards specific scenarios related to Ambient Assisted Living (AAL) for elders.

We advocate that, in the ambition towards open access, such an architecture and data sets, an important step is to push the state of the art in smart home technology. By providing other researchers with an easy way to implement and integrate data from different sources, we believe that data collection is made easier. The collected data sets showcase the modularity of the system and the possibility to implement it with different focuses in mind while providing labeled and easy-to-use data for researchers interested in AAL and activity monitoring for elderly people.

To outline our contribution, we have presented the E-care@home system in Section 3, where we focus on its building blocks from the hardware layer (e.g., the sensor network) to the software modules (e.g., context-recognition, person counting, and configuration planning modules). Further details including our test bed called Ängen, the data-collection process, and the different labelled data sets are given in Section 4.1 and Section 4.

Public access to software and data sets can be found under the following addresses.

- Public GIT repository: https://gitsvn-nt.oru.se/uwe.kockemann/ecare-pub
  Software modules and E-care@home cookbook (detailed guide to setting up and using E-care@home modules)
- Database: https://ecaredb.oru.se (user name and password: ecare-pub)
  Public access to data sets described in this paper

## 2. Related Work

The Internet of Things (IoT) is a paradigm in which many everyday objects are equipped with sensing and actuation components as well as processing abilities and can communicate with each other to offer a service to a user in a transparent way. Homes (or more generally living environments) equipped with such devices and various sensors are referred to as *smart homes*. Many IoT systems and smart homes have been developed during the past decade with various purposes. Whether the goal is to optimize energy consumption [1,2], to increase the user’s comfort by activating or deactivating devices [3], to monitor air quality [4], or to monitor and assist elderly users [5], all Smart Home systems have several components in common: (1) a hardware platform to gather data (environmental or wearable sensors), (2) a set of applications to gather the data, and (3) a repository to store the data. Recently, more and more systems also embed reasoning modules to perform activity and context recognition.

Some work has been carried out to propose architectures for generic data-collection systems that can be installed and used by a wide range of users without training. For instance, this is the case of the CASAS architecture (and associated data sets) [6], which includes a hardware platform containing motion sensors, door sensors, and temperature sensors; a middleware in the form of a publish/subscribe architecture to gather the data; and several reasoning modules for activity recognition, activity discovery, and energy usage analysis. The system is focused on single-user monitoring in smart homes, and the activities recognized are very varied (e.g., *cook*, *personal hygiene*, and *phone*) and are recognized with very different rates of success; the recognition of the *phone* activity is, for instance, much less successful than the *cook* activity. The E-care@home system presented in this paper is close to the CASAS system, as it aims at providing potential users with a modular architecture to create an IoT infrastructure as well as at providing collected data sets. It differs from CASAS by two factors. The first is more focused toward home care and monitoring of elderly users and, for this reason, includes wearable sensors to collect physiological data. Second, it includes a reasoning module capable of performing person counting that will allow the use of the system even when several occupants are present (see Section 3.4.2 for the complete description of the module).

Other pieces of work focus on providing annotated data sets for researchers, both for single and multiple occupants. The DOMUS dataset [7], for instance, includes data for many different types of sensors (e.g., electricity and water counter, presence detector, temperature, and air quality), and an API to interact easily with the data. It does not contains any labeling for the activities performed (the ground truth). The ARAS data set [8], however, contains the ground truth for 27 different activities in addition to the data from 20 binary sensors. Similarly, the SPHERE data set, which was provided for an activity recognition contest, contains annotations for 20 different activities [9]. More recently, the ContextAct@A4H data set [10] provides researchers with data from 17 sensors, including not only the most common sensors in smart homes such as pressure sensors, contact sensors, electricity, or water consumption sensors but also more rare sensors such as switch status sensors, CO_2_ devices, as well as a wearable indoor positioning system. It also includes annotations for 7 different activities.

Due to the difficulty of gathering data in smart home settings, simulators have been developed to provide realistic sensor data [11,12] and to allow algorithms to become much easier to evaluate on large amounts of data. A part of the work carried on within the E-care@home research environment focused on this problem and proposed an approach combining constraint-based planning with agent-based simulation to produce realistic data sets [13]. This work is still ongoing and not part of the E-care@home system yet and is therefore out of the scope of this paper.

## 3. The E-care@home System

**Figure 1:**
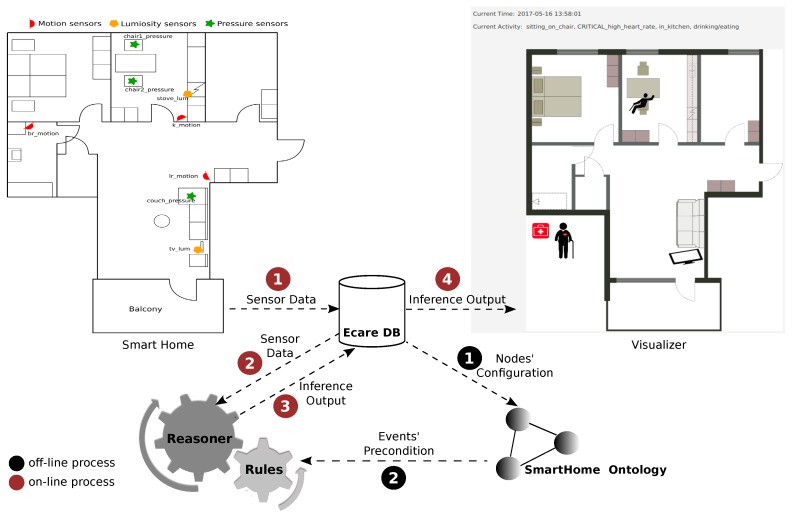
An abstract view of the context recognition system in E-care@home: The system is initialized offline based on the black-labeled links. After the initialization, the running system relies on communication links among different modules shown in red labels.

The E-care@home system (https://gitsvn-nt.oru.se/uwe.kockemann/ecare-pub.git) is an interconnected set of hardware and software modules capable of collecting and storing sensor data. The system has been designed with one main challenge in mind: semantic interoperability. The overall system is presented in Figure 1.

At the center of the system, the E-care@home Database and the ontology allows for semantic interoperability by integrating and reasoning over data coming from various sources, including not only sensors but also a priori knowledge and prior reasoning results. Data from sensors are stored in the database and used to populate the ontology, which contains a prior knowledge, and is then combined with a semantic reasoner to interpret the data on a semantic level. Inference outputs are in turn stored into the database as labelled temporal intervals, which allows to use them in future reasoning, combined with additional sensor data. For instance, the reasoner could derive the activity *eating* from a previously derived activity *cooking* and sensor data indicating that the user is now sitting at the kitchen table. In addition to data and reasoning output, the E-care@home Database can contain the ground truth of performed activities, also stored as labeled temporal intervals. This ground truth can be used to evaluate the results of the different reasoning modules.

The E-care@home system can be extended with new sensors and other data sources as long as these sources output their data in the E-care@home Database format (described in Section 3.1).

**Figure 2:**
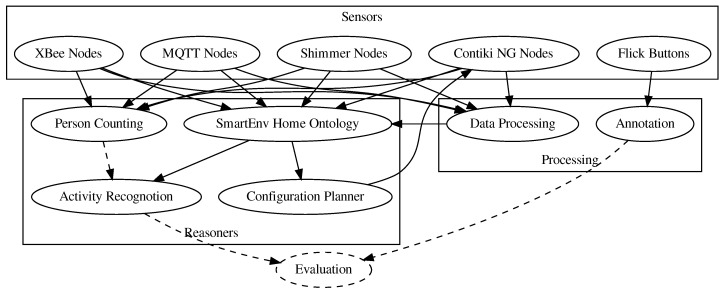
Data flow between all modules in the E-care@home system: Modules are subdivided into three classes for sensors, processing, and reasoners. In the actual system, all data goes through the database.

Figure 2 shows the hardware and software modules of the E-care@home system as well as the flow of data between them (To be able to show the interconnection between the modules, the database is not included in the figure.). Dashed elements correspond to future work in which we intend to evaluate such as the combination of person counting and activity recognition.

In the following, we will describe each of the modules presented in Figure 2, starting with the central E-care@home Database. Each module is described conceptually and focuses on the role of this module in the whole architecture. Performance analysis and results about the reasoning modules are out of the scope of this paper and can be found in dedicated publications (referenced in the relevant sections). Complete instructions on software setup and usage (as well as the software itself) can be found in the cookbook in our public GIT repository. In addition to the modules described in this paper, the GIT repository contains a set of software components used for easy visualization, evaluation, and configuration of some of the components.

### 3.1. The E-care@home Database

**Figure 3:**
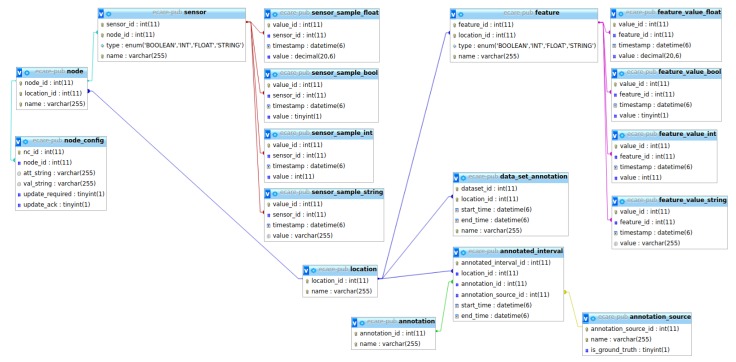
Table diagram of the E-care database.

The diagram of the database is presented in Figure 3.

Our data-related infrastructure, which is designed to play the role of data repository, was decided to be separated from the semantic-related infrastructure. We use MySQL as an open-source *Relational Database Management System (RDMS)* due to its high availability and on-demand scalability. This allows us to write data collection and processing software as well as AI reasoning modules independent of the origins of data. This is useful when dealing with a heterogeneous collection of sensor systems based on different types of sensor networks.

Our database, called the E-care@home Database, associates all information to a location, which is typically a specific smart home. Locations contain sets of nodes, and each node contains one or more sensors, all of which are stored in the database. The actual data produced by sensors is stored in various sensor value tables (for integers, Boolean values, floating point numbers, and strings). Names of nodes and sensors adhere to a format that allows us to automatically populate the E-care@home ontology with information regarding the sensor placement and what it measures. Our naming convention makes it possible to compose names of locations, nodes, and sensors attached to node to build a coherent name that resembles a topic as used in MQTT (https://mqtt.org/) and similar systems. Table 1 lists a number of sensor names used in E-care@home. For instance, the sensor name *livingroom/couch/pressure* is parsed into three sub-strings indicating the specific location in the smart environment where the sensor is located (*livingroom*), the object that the sensor monitors (*couch*), and the property of interest that also indicates the type of sensor (*pressure*), respectively. Each sub-string will be added as an instance of a elevant class in the ontology to prepare it for high-level reasoning tasks about events and activities.

In addition to sensor data, we also allow the storage of features. Features are essentially the same as sensors except that they are not connected to a sensor node. Features are used for processed data such as filtered sensor data or inferred values that are not directly connected to a sensor (e.g., tiredness).

Sensor and feature information is stored in the form of time-stamped values. In addition, we use annotations to store information about temporal intervals. Each annotated interval contains a start time, an end time, an annotation, and an annotation source. Keeping track of sources allows us to distinguish between ground truth and annotations that result from reasoning systems (e.g., activity recognition).

### 3.2. E-care@home Sensing Modules

In this section, we describe the various software and hardware sensing modules that contribute to the E-care@home system. Each of these modules provides a specific functionality to the overall E-care@home system. This modular approach makes the system easy to extend and use only parts. The purpose of modules are (a) collect data from sensors, such as sensors connected to XBee Wireless networks, Contiki IoT nodes, or Shimmer wearable sensors; (b) to process data by applying filters, calculating thresholds, etc.; (c) to label time intervals of data to mark activities, ground truth, or data sets; and (d) to reason about sensor data in a semantic context (e.g., considering where and how a sensor is installed, its measurements will have a context-dependent meaning.)

We hope that, by making these modules available open source, it will be easy for others to connect their own sensor suites, reasoners, or processing solutions to work with collected and streamed data.

#### 3.2.1. Contiki-NG Sensor Nodes

E-care@home deployments rely on different types of IoT sensor devices that run the Contiki-NG operating system. The primary use case for these devices is an infrastructure of environmental sensors, capable of measuring a variety of phenomena such as presence, temperature, humidity, and pressure. A key aspect of such an installed infrastructure is the reliance on batteries as the primary energy source. Hence, the infrastructure must run in an energy-efficient manner, as frequent battery replacements would come with a high-maintenance cost. IoT nodes using Contiki-NG also allow configuration during run-time, which is of interest for configuration planning (see Section 3.4.3), where we would like to dynamically decide sensor configurations (on/off, frequency, etc.) based on the context (e.g., users at home and high fall risk).

Contiki-NG is an open-source operating system supporting standard protocols for low-power wireless IPv6 communication and is being developed mainly by researchers at RISE (Research Institutes of Sweden) and the University of Bristol. To support energy-efficient and reliable communication in the E-care@home system, we have developed parts of Contiki-NG’s implementation of the IEEE 802.15.4 Time-Slotted Channel Hopping (TSCH) protocol [14]. For the collection of sensor data and run-time assurance metrics, we use the standard Lightweight Machine-to-Machine (LwM2M) protocol [15], which operates atop the Constrained Application Protocol (CoAP). To extend the possible reach of the deployment, the Contiki-NG sensor nodes are capable of multi-hop routing by using the standard RPL (Routing Protocol for Low-Power and Lossy Networks) routing protocol with enhancements for bidirectional traffic [16], which is beneficial for LwM2M.

The main hardware platform employed for the Contiki-NG sensor nodes is the Zolertia RE-Mote. This platform is equipped with a Texas Instruments CC2538 System-on-Chip with a 32 MHz ARM Cortex-M3 processor, 512 kB flash memory, and 32 kb RAM. The RE-Mote also has two different IEEE 802.15.4-compliant radio transceivers: one for the 2.4 GHz band and one for the 863–950 MHz bands. A variety of environmental sensors are available for the platform, such as Sensirion SHT25 humidity and temperature sensors, TSL2563 ambient light sensors, and PIR (Passive InfraRed) motion sensors. In case there is need for other types of sensors in the future, the platform can be extended through digital and analog I/O interfaces. Because the E-care@home system uses standard protocols such as TSCH and LwM2M for communication, also other platforms supported by Contiki-NG can be added to smart-home deployments with low effort. The platform-dependent part of the E-care@home application layer is implemented in a separate module and contains mainly functionality for answering read requests for its different types of sensors.

Using the Contiki-NG based nodes, we also carried out some initial work on run-time assurance [17] in which we collected network-related statistics (such as success rate of package transmissions) to allow assessing the status of the IoT network. Run-time assurance is an important aspect of long-term autonomy and needs to be considered for any system that is supposed to operate for an extended amount of time with little to no supervision.

#### 3.2.2. XBee Sensor Nodes

Our second sensor module collects data from a network of XBee (https://www.digi.com/xbee) wireless sensor nodes. These nodes are relatively simple and cannot be programmed. To use this module, we require a border node connected to the machine that collects the data. To configure it, we provide a mapping from XBee addresses and analog-digital ports to a sensor name and value type in the database. The node entry in the database corresponds to the XBee’s network address.

In the Ängen apartment, we use XBee wireless sensor modules to collect data from motion sensors, pressure sensors, and contact sensors. Motion sensors cover ambient motion in each room and are used to detect motion next to the bed (see Section 4). Pressure sensors are used under the bed, couch, and chairs. Contact sensors are used to detect whether the fridge, microwave, and a cabinet in the kitchen are open or closed.

#### 3.2.3. MQTT Module

Many IoT devices can easily be configured to send data to a specific topic on an MQTT (https://mqtt.org/) server. Other software modules can then subscribe to these topics in order to receive data as it arrives. This achieves the same level of decoupling as our E-care@home Database but without storing data.

Our E-care@home MQTT module provides a simple way to map MQTT topics to nodes and sensors in the E-care@home Database. As a result, we can easily access and store any data made available in this way. As for most of our modules, configuration is handled in a central JSON (JavaScript Object Notation) file which links *MQTT (Message Queuing Telemetry Transport)* topics to sensors in the E-care@home Database.

Out of the three presented modules, this is probably the easiest to set up by other researchers, since it can simply connect to existing infrastructure.

#### 3.2.4. Shimmer Wearable Sensors

In order not only to recognize general activities performed by humans but to also enable real-time monitoring of physiological parameters and following of health trends, wearable sensors are needed. By wearable sensors, physiological parameters such as heart rate, oxygen saturation, electrocardiogram (ECG, the electric activity of the heart), and electromyogram (EMG, muscle activity) can be recorded in addition to motion parameters such as acceleration and position (measured by accelerometers, gyroscopes, and magnetometers for physical activity recognition, fall detection, and fall prevention). To achieve this, we have used the Shimmer platform, which provides wireless data acquisition and real-time monitoring of, e.g., heart rate, respiration rate, oxygen saturation, ECG, EMG, skin temperature, skin conductance, and body movement. Several APIs such as MATLAB, C#, Java/Android, and LabView API are tested and could be used to develop software in order to stream health parameters directly to any Android/computer device and to forward it further to the E-care@home databases (RDMS MySQL), both locally and in the cloud. Several health parameters can be measured simultaneously with the limit of Bluetooth communication, depending on sampling frequency, signal types, and processor of the mobile device. Maximum battery consumption is achieved by the ECG shimmer node around 10 h; however, it can adjust by controlling the writing/transmitting alternatives.

Shimmer3 devices are equipped with an MSP430 microcontroller (24 MHz, MSP430 CPU), a Bluetooth radio of RN-42, an 8 GB micro-SD card, and a 450 mAh rechargeable Li-ion battery. The Shimmer3 supports the class 2 Bluetooth 2.1 + Enhanced Data Rate (EDR) module, which is classified as Bluetooth classic. The maximum sampling frequency of the Shimmer3 is 8 kHz, which stands for sensing frequency. The wireless transmission limits the sampling frequency as there are limitations with the data rate and packet length. There is no specified limit on the maximum sampling frequency attainable in conjunction with high data delivery. In our tests, we achieved a high data delivery with a 500 Hz sampling frequency. When the sensor is able to operate with high sampling frequency but the Bluetooth is unable to transmit with the same sampling frequency, one must either buffer some of the data in the Shimmer’s memory and send a chunk of data with a lower sampling frequency over the wireless link or one must simply reduce the sensing sampling frequency.

Two main Shimmer units were used for the experiments; (i) *Inertial Measurement Unit (IMU)* for movement detection and (ii) ECG for heart rate monitoring. The IMU gives nine degrees of inertial measurement by encompassing the accelerometer, gyroscope, and magnetometer.

### 3.3. Processing Modules

This section discusses modules developed for data processing, labelling, as well as visualization.

#### 3.3.1. Data Processing

The data processing module provides a way to apply functions to streams of data. Each data processing function maps a sensor or feature to a single output feature. During run-times, each function will check for new input data periodically and add new feature values to the database as they are computed.

Currently, this module provides the following functions:

- compute the median of a time series over a fixed window size;
- compute the change between subsequent values of a feature/sensor time series;
- apply a threshold to a time series (e.g., to convert from pressure sensor data of a couch to couch usage data); and
- calculate the average frequency of data points based on their time stamps over a fixed window size.

Configuration is handled via the central E-care@home JSON file, where one can specify which existing filters to apply to any data stream in the database.

#### 3.3.2. Data Labeling

Collecting the *ground truth* when gathering sensor data is a very important and complicated process. The ground truth is mandatory to test and evaluate a system. However, it implies that the data must be annotated manually, either in real time or a posteriori. Most of the systems existing currently require the user to manually annotate their activities in real time by providing dedicated graphical user interfaces [8], logbooks, or audio-based systems capable of recognizing vocal commands [18]. Some other systems are using video cameras [19] or raw sensor data with the help of a visualizer [20].

In the E-care@home system, we use real-time annotation with pervasive connected buttons (The Flic Button https://flic.io). Each button is associated to a label and placed in a strategic position in the environment. When the environment enters a given state of interest or when the user engages in an given activity, they press the corresponding button and the corresponding timestamp is stored by the software on which the button is connected. When the environment leaves the state or when the user finishes this activity, they press the button a second time, the corresponding timestamp is recorded again and the label is sent to the E-care@home Database with start and end timestamps. For instance, if we are interested in labeling the activity “cooking”, we would place a connected button in the kitchen area. When the user starts and stops cooking, they press the button which is located in the area where the cooking usually takes place. This solution makes the real-time labeling less cumbersome than other real-time solutions in the literature. Indeed, the user is not required to carry anything with them as the necessary pieces of equipment are distributed in the environment where the user will likely need them. The user is not required either to remember any specific command, as all the labeling is done with a simple press on a button.

The labels are decided through a specifically designed software, the E-care@home Labeling Software. Any type of label can be added, which makes the solution generic and adaptable. The same solution can be used to label activities, to count the number of persons currently in the home, or to check specific people in/out. In addition to configuring the label, the E-care@home Labeling Software also allows the user to monitor in real time which label is currently on (i.e., the button has been pressed once and the activity or state is ongoing) and to ensure that the labeling is being carried out correctly. It also allows the user to delete or modify a previously recorded label in case of a mistake and to add a label manually if the user forgot to press the buttons.

This system has been tested both in controlled and uncontrolled environments. In controlled environments, predefined scenarios were acted by test subjects, who also were required to label their activities. In the uncontrolled environment, the system has been placed in the home of a user for 2 weeks as part of an actual data collection process.

#### 3.3.3. Data Visualization

Visualization is important for testing during system development and to allow identifying patterns when data is collected. This module visualizes data as it is collected into the E-care@home Database. It essentially takes data from the database and forwards it to Python matplotlib (https://matplotlib.org/). It is configured by providing a mapping from sensor names to a plot and its configuration.

### 3.4. E-care@home Knowledge-Driven Reasoning Modules

The E-care@home system contains three reasoning modules (the activity recognition module, the person counting module, and the configuration planner) and the SmartEnv ontology. The three reasoning modules rely on a knowledge-driven context recognition (CR) approach. By knowledge-driven, we refer to an approach where the activities and events of interest are recognized by capturing contextual information based on prior domain knowledge and then by performing reasoning over the contextual information. In our case, the prior information is encoded in the SmartEnv ontology.

#### 3.4.1. SmartEnv Ontology and Activity Recognition

We have developed an ontology called SmartEnv [21] as the knowledge model of the E-care@home system. The SmartEnv ontology relying on upper level ontologies allows us to model a smart environment equipped with heterogeneous sensors in the form of objects, their features of interest, possible activities/events, and the other spatiotemporal aspects of the environment.

In SmartEnv, activities are defined as subclasses of the class *Event*, which as such is categorized into two classes of *Manifestation* and *ComplexEvent*. A manifestation refers to an activity or a change in a given environment that can be recognized directly from sensor data (e.g., pressure sensor under the couch is triggered). Although a manifestation can reflect an activity (e.g., sitting on the couch), there are other situations that are defined as a combination of several manifestations (i.e., preconditions). The class *ComplexEvent* is defined in SmartEnv to model such situations. Each complex event is defined as a combination of several manifestations as its preconditions with specific temporal relations. The reasoner, which is continuously receiving data (or changes in the data in the form of manifestations) from different sensors, is able to recognize predefined complex situations in real time, independent of their occurrence frequency, provided that their preconditions are met.

The initialization process of the E-care@home system is composed of two offline processes shown in Figure 1 with black-labeled links. The first process is about populating the ontology based on the information given in the database. This information includes the location of the smart home (i.e., the name of the environment), the names of the hardware nodes (holding sensors), their sensors, and finally the feature of interests (i.e., the combination of an object and its property) observed by each sensor. The second offline initialization process is about generating logic programs based on activities and events defined in the ontology (SmartEnv) and are going to be read by the reasoner during the monitoring process. The SmartEnv ontology is publicly available and can be found here: https://w3id.org/smartenvironment/smartenv.owl.

The monitoring process is divided into several subprocesses shown in red-labeled links in Figure 1. The process starts by sensing the environment through sensors deployed in the environment. The sensors data labeled with timestamps is directly recorded to the database. The context-recognition process concurrent with the sensing (observation) process starts by fetching the recently recorded data in the database (red link number 1 in Figure 1) and by feeding the reasoner with the data (red link number 2 in Figure 1). Given the data, the reasoner which is already initialized with logic programs generated from the ontology results in time intervals which are labeled with the name of activities, events, or whatever inferred by the reasoner.

More specifically, the logic programs contain logic rules that represent events (i.e., complex events) in terms of their preconditions (defined in the ontology) as follows:

$$\begin{array}{c} event\left(E,t\right)\;:-\;eventCondition\left(C_{1},t\right),\;eventCondition\left(C_{2},t\right),\;...,\;eventCondition\left(C_{n},t\right). \end{array}$$

As shown in the following, each *EventCondition* predicate also represents a manifestation and its temporal relations w.r.t. a timestamp at which the event is expected to occur. The *lowerBound* and the *upperBound* values indicate the preferable temporal distance between an event and its preconditions:

$$\begin{array}{c} eventCondition\left(C_{1},t\right)\;:-\;manifestation\left(O_{1},P_{1},S_{1},T_{1},T_{2}\right),\\ \;t-lowerBound\;<=\;T_{1},\;T_{1}<=\;t-upperBound,\\ \;t-lowerBound\;<=\;T_{2},\;T_{2}\;<=\;t-upperBound,\\ \;T_{1}\;<=\;T_{2}. \end{array}$$

Each event is defined in the form of three different logic rules indicating starting, continuation, and ending conditions of the event. the following examples show the three rules related to the event (or activity) *sittingOnCouch*. To simplify, we assume that there is no temporal distance between the event and its precondition:

(1) $$\begin{array}{ccc} \mathrm{starting rule}: & & \\ & \;event(sittingOnCouch,\;t)\;:- & \\ & & \;situation(couch,pressure,true,t),\;not\;event(sittingOnCouch,\;t-1). \end{array}$$

(2) $$\begin{array}{ccc} \mathrm{ending rule}: & & \\ & \;event(sittingOnCouchEnd,\;t)\;:- & \\ & & \;situation(couch,pressure,false,t),\;\;event(sittingOnCouch,\;t-1). \end{array}$$

(3) $$\begin{array}{ccc} \mathrm{continuation rule}: & & \\ & \;event(sittingOnCouch,\;t)\;:- & \\ & & \;event(sittingOnCouch,\;t-1),\;not\;event(sittingOnCouchEnd,\;t). \end{array}$$

As shown in Figure 1, the labeled intervals (during which the occurrence of an event has been reported) as the result of the reasoning process are recorded in the database (red link number 3). Once the inference results are generated and recorded in the database, the other modules such as the visualizer are updated in order to visually represent the current situation of the smart home (red link number 4).

The three rules representing conditions under which a complex event can be recognized are able to always be triggered as long as the value of their parameter t is matching with the current timestamp read from the sensor data. The reasoner relies on an incremental AnswerSet solver called Clingo [22]. Clingo controls the value of parameter t, which grows incrementally to enable the reasoner to always reflect the current state of the environment based on sensor data. Further details of the reasoning process can be found in Reference [23].

#### 3.4.2. Person Counting

In real-life situations, it is common that more than one person will be present in a house. Whether it is because several persons are living together or because the occupant is receiving visitors, reasoning systems need to take into account this possibility. While commercial solutions exist for counting persons, they are not adapted to smart homes, either because they are targeted for crowd counting [24,25] or because they use vision-based sensors and are therefore too intrusive [26].

**Figure 4:**
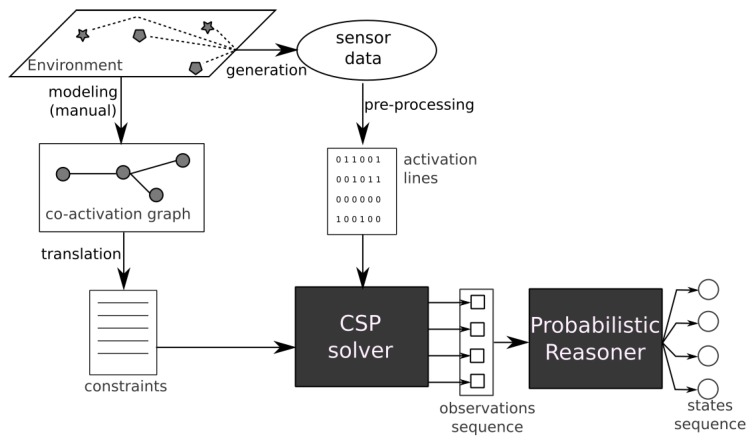
Overview of the person counting module’s architecture. From Reference [27].

The E-care@home system includes a person counting module capable of estimating the number of persons currently present in the smart home using only environmental sensors. This module, shown in Figure 4, combines a Constraint-Satisfaction Problem (CSP) solver with a Hidden Markov Model (HMM) to provide a robust estimate of the number of persons present in the environment based on the sensors activated in the environment.

The person counting module uses as inputs *activation lines*, which are observations of *Features of Interest (FoI)*. A feature of interest is the abstract representation of a real-world event that indicates the presence of a person. For instance, *couch occupancy* is an FoI, as are *door opening* and *tap opening*. Features of interests can be directly measured from sensors (e.g., couch occupancy is directly measured from a pressure sensor situated under the couch) or after some preprocessing (e.g., door opening requires to detect the moment when the door sensor change values as it is the action of opening the door and indicates the presence not the door being open). An *activation line* is a mapping from the sensor data to {0,1} indicating for each FoI if it is activated (1) or idle. In addition to the activation lines, the person counting module requires a graph called *co-activation graph*, indicating which FoI can be activated simultaneously by the same person. For instance, a motion sensor in the living room and a pressure sensor under the couch can be activated by the same person at the same time, and therefore, their corresponding FoIs would be linked in the graph. On the contrary, a single person should not be able to activate a pressure sensor under the couch and a motion sensor in the bathroom at the same time.

The person counting module uses a two-step reasoning process. First, a *Constraint Satisfaction Problem (CSP)* solver is applied on a single activation line, providing the minimum number of persons required to provide the observed activation line. Second, a series of outputs from the CSP solver is sent to a probabilistic reasoner (a Hidden Markov Model) which estimates, according to previous information, the actual number of persons in the environment. The final estimation is stored in the E-care@home database as a temporal interval and can be used by other reasoning modules.

For more details about this module and experimental results, we refer the reader to Reference [13].

#### 3.4.3. Configuration Planning

For long-term smart home installations context- and goal-sensitive smart home configuration becomes an important issue. Sensors should be on full-alert during critical periods (e.g., when a fall is most likely to occur) and may be turned to minimal usage in others (e.g., when the user is not at home). The purpose of configuration planning in E-care@home is to automatically decide how to configure sensors in a given context. Examples of context are as follows:

- High likelihood of fall
- User not at home
- Nurse is visiting

Configuration planning [28]. is the integration of AI task planning with information goals and dependencies. We developed a temporal configuration planning approach [29] that addresses this problem by linking context to information goals.

Given a temporal constraint network describing the context of our system (e.g., high fall likelihood between 8:00 a.m. and 8:15 a.m.), each context has a set of information goals. Information goals can be satisfied by following information links of the following form:

$$I\overset{c}{\underset{\mathrm{TR}}{⟶}}O$$

where each link specifies an input *I*, an output *O*, a cost c, and a task requirement TR. This model captures information sources such as sensors as well as modules that require information.

In a high fall-risk context, an information goal could be *fall*, which may be satisfied by a link such as the following:

$$\left\{(\mathrm{video-feed}\;\mathrm{camera-}1)\}\overset{c}{\underset{\left\{(\mathrm{on-user}\;\mathrm{camera-}1)\right\}}{⟶}}\{\mathrm{fall}\right\}$$

When the user is not at home, this information goal may be switched to *user-at-home* which may use a link such as the following:

$$\left\{\mathrm{motion-in-apartment}\}\overset{c}{\underset{\left\{\mathrm{motion-sensor-frequency}:=0.1\mathrm{Hz}\right\}}{⟶}}\{\mathrm{user-at-home}\right\}$$

Task requirements allow to specify actuation requirements such as focusing a sensor on an object or sending a robot with specialized sensors to a specific location. This model covers cases (such as robotics) that are beyond the focus of this paper. In our previous work, we considered two solutions to this problem. The first one solves information goals by mapping them to goals of a temporal interval planner. The second approach models information goals as a separate subproblem that is solved by using constraint processing. The details of this approach can be found in the referenced paper.

The work of integrating configuration planning into the E-care@home system is still ongoing. We did some initial work on controlling Contiki-NG IoT nodes with the same nodes used by the approach described above [30]. In addition, we studied ways to automatically generate configuration planning problems from our SmartEnv ontology [31], which is also used to interpret sensor data (as explained in Section 3.4.1). In ongoing work, we are investigating approaches to modify the network traffic schedules of the IoT network by utilizing context-dependent requirements available to the AI planner.

The planning system we use, Spiderplan (Available open-source under spiderplan.org), is quite versatile and can be extended to control any device found in a smart home (as long as it can be controlled by custom software).

## 4. Data Sets

The E-care@home project focuses on assisted daily living for elderly people, especially presenting dementia or comorbidities. The types of data sets needed and collected were therefore targeting relevant scenarios with regard to this application domain.

During this project, we conducted three different data collection campaigns. The first one (Section 4.2) aimed at gathering everyday life data about a person living alone in their apartment and focused on activities relevant for the E-care@home project (the full list of activities is provided in Table 2). The second campaign was targeted toward sleeping activity, which is of great importance for elderly people as sleep disturbances are a strong early indicator or dementia [32]. Therefore, we focused on detecting the presence of a person in bed, their possible movements in bed, as well as the fact that they are sitting on a side of the bed (which indicates that the person is not sleeping, even though standard pressure sensors might detect the person being in bed). Finally, the third campaign targeted the person counting module specifically and aimed at providing labelled data considering more than one or two persons in the environment. Indeed, existing data sets consider either only one person or a “having guests” activity, which does not discriminate between the number of persons present. Table 1 presents the list of sensors used for the different data sets and their sending frequencies.

All data sets are fully labeled thanks to the process described in Section 3.3.2 and are stored in the E-care@home database as annotated intervals. The first data set was recorded in a personal home, temporarily equipped with sensors (the home has not been build as a smart home and sensors were installed only for the duration of the data-collection campaign). The second and third data sets were recorded in our Ängen facility, which we describe in Section 4.1. All the data sets are available in the public version of the E-care@home database.

### 4.1. Data Collection at Ängen

**Figure 5:**
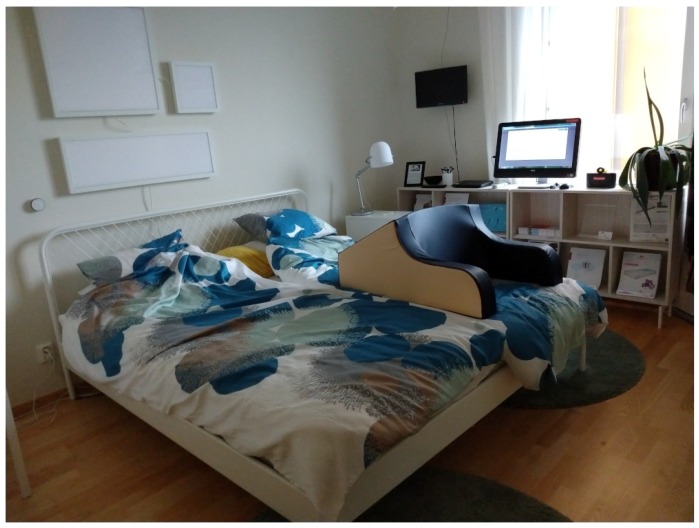
The bedroom of the Ängen research and innovation apartment.

Located approximately 2 kilometers from the center of Örebro, Sweden, is a large elderly and health facility. The facility contains a number of different types of living accommodations, a health clinic, a daily rehabilitation center, and a family center within the same block. This facility called Ängen also contains two apartments which function as labs. In these labs, technology can be deployed and user testing can be performed. The apartments are well equipped for testing purposes, allowing for experiments to be monitored via video recording or other means for ground-truth collection. Furthermore, the apartments have been used for testing and deployments in a number of European Projects including RobotEra, GiraffPlus, Sappho, as well as E-care@home. Figure 5 shows the bedroom of the apartment.

### 4.2. Real Apartment Data Set

**Figure 6:**
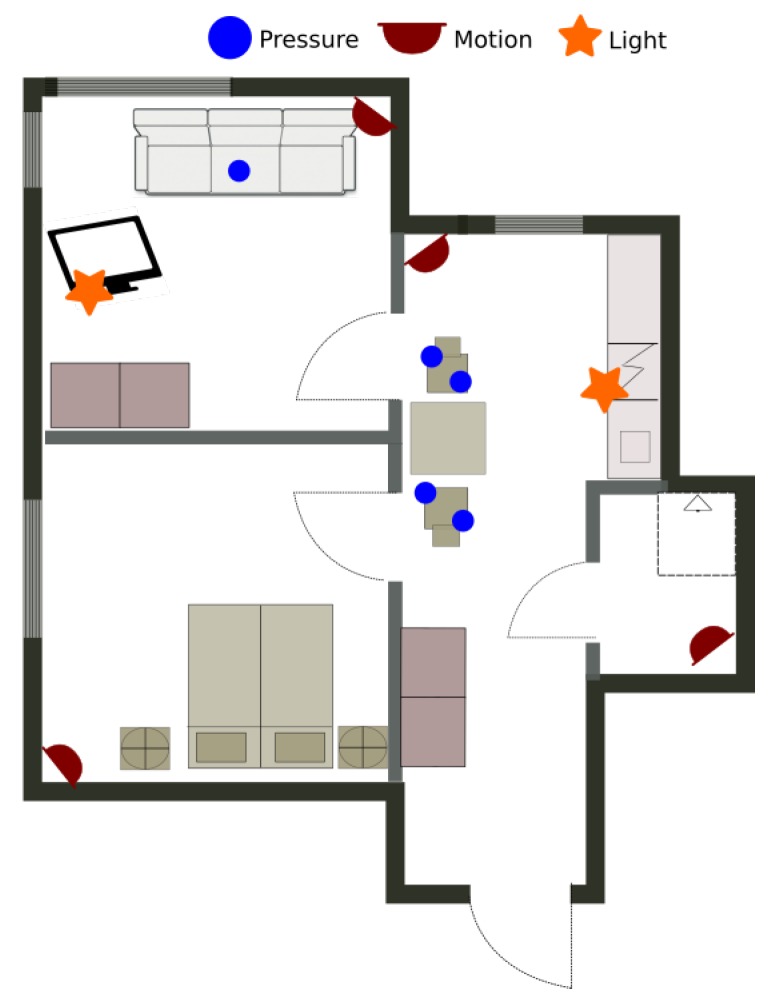
The smart apartment and the position of the sensors for the real apartment data set.

The first data set is gathered from a single inhabitant apartment equipped only with environmental sensors. The layout and the position of the sensors are presented on Figure 6. The data was gathered constantly during 10 days from 24 December 2017 11:00 until 3 January 2018 20:00, when the user has also labeled some of the activities relevant for E-care@home. Table 2 illustrates the activities labeled by the user. The data set is annotated in the database as *ORU/Home1/01*.

**Figure 7:**
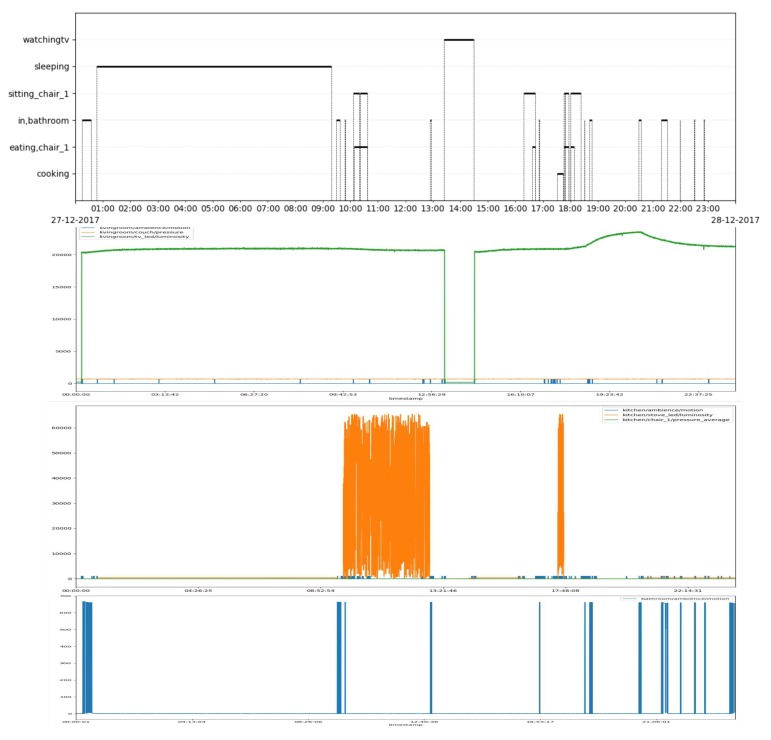
An example of the data gathered from the campaign: The top row shows the activities as labelled by the user, the second rows shows activation from the sensors in the living room, the third row shows activation from the kitchen, and the last row shows activation from the bathroom. Please note that the TV LED is lit when the TV is off while the oven LED is lit when the over is on. We can see from row 3 that the oven LED is on between 09:00 and 13:00, while the cooking activity has not been labelled, which might indicate that the user turned the oven on by mistake and forgot about it.

Figure 7 shows as an example the timeline of the activities labelled by the user for one day, together with the sensor activation patterns observed during this day.

### 4.3. Bedroom Data Set

This data set was collected with a focus on activities related to getting in, lying in, and getting out of bed. As before, we use motion and pressure sensors to measure motion in the room as well as under the bed and pressure under the different feet of the bed. We also included a set of wearable sensors for accelerometer and heart rate data.

It includes both environmental and wearable sensors. The data has been collected in the research and innovation apartment Ängen, where the user (the agent) was a researcher and not a permanent inhabitant.

**Figure 8:**
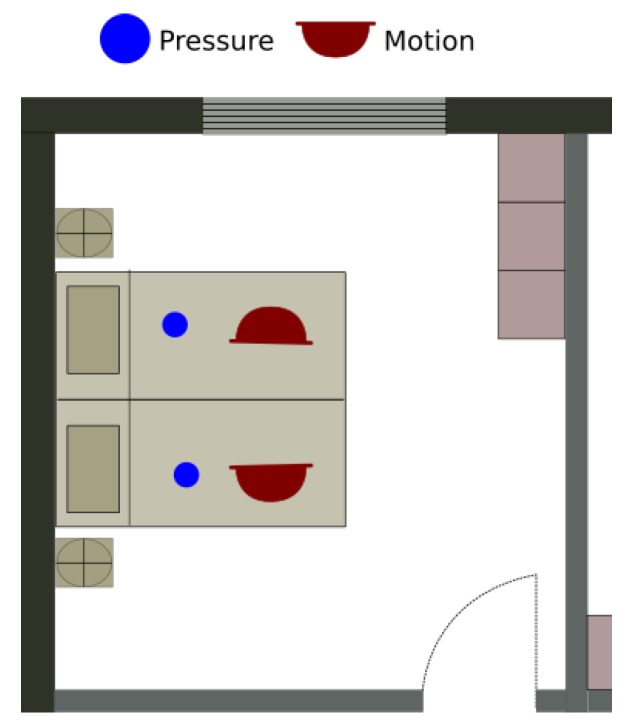
The type and locations of sensors used in the bedroom data set.

A total of seven sensors are installed in the apartment, four of which are environmental and three of which are wearable sensors that are embedded in three Shimmer nodes worn by the user. The layout of the sensors installed in the bedroom is shown on Figure 8. The details of the wearable sensors are given in Table 3. The data set is annotated in the database with *ORU/Angen/BedroomActivities/01*.

The data was gathered at daytime for approximately five hours from 21 February 2018 10:22 until 21 February 2018 15:42. Table 4 illustrates the list of the activities (from the table *annotation* in E-care@home Database) labeled by the user.

### 4.4. Person Counting Data Set

**Figure 9:**
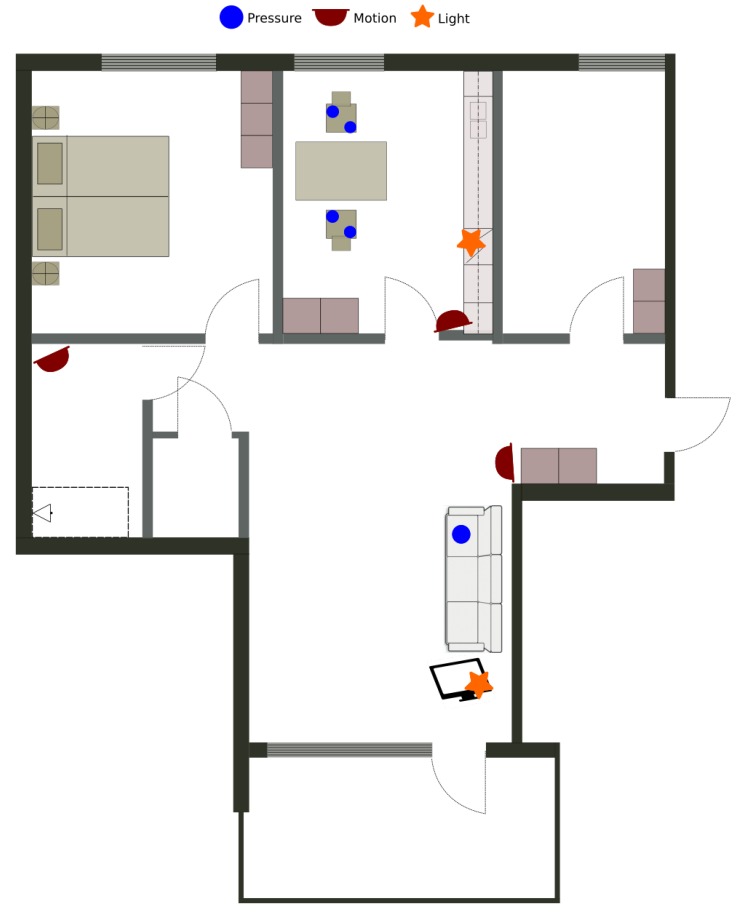
The Ängen smart apartment and sensors used for the person counting data set.

To evaluate our approach for person counting on real data, we recorded a series of data sets in our Ängen smart apartment, in which we play through typical multi-person scenarios. The layout of the apartment and the location of the sensors is presented in Figure 9. The actual number of occupants are annotated as annotated intervals, with ids and names presented in Table 5.

For each scenario, three occurrences of these scenarios have been recorded with different actors and slightly different timings for the actions. The timestamps corresponding to each instance of each scenario in the database are presented on Table 6. Section 4.4.1, Section 4.4.2 and Section 4.4.3 describe the different scenarios and the corresponding data set label.

#### 4.4.1. Scenario 1: Visit

The first scenario is a visit in a smart apartment occupied by one person and has been conducted as follows, with P1 referring to the usual occupant of the apartment and P2 referring to the visitor:

1. P1 enters the apartment and sits on the couch in the living room
2. P2 enters the apartment and goes in the living room (without sitting)
3. P2 goes in the kitchen and prepare tea
4. P1 goes in the kitchen and sits on a chair
5. P2 sits on the other chair
6. After a certain amount of time, P1 gets up and goes in the living room to sit on the couch
7. Meanwhile, P2 gets up and cleans the kitchen
8. P2 leaves the apartment
9. P1 leaves the apartment

#### 4.4.2. Scenario 2: Three Occupants

The second scenario considers three occupants and has the following steps:

1. P1 enters the apartment and goes to the kitchen
2. P2 enters the apartment, goes to the living room, and sits on the couch
3. P3 enters the apartment and goes to the kitchen
4. P1 goes to the living room (without sitting)
5. P1 goes to the bathroom
6. P3 leaves the apartment
7. P1 comes back to the living room
8. P1 leaves the apartment
9. P2 leaves the apartment

#### 4.4.3. Scenario 3: Four Occupants

The third scenario has been designed with a similar goal as for the second one: providing different activation patterns for four occupants. However, this scenario also has one main difference from the two others: the scenario starts and finishes with at least one person in the apartment already. This is meant to test the ability of a person counting system to derive the number of persons actually present even when no entrance activation has been detected.

This scenario has been conducted as follows:

1. P1 is present in the apartment
2. P2 enters the apartment, goes to the kitchen, and sits on a chair
3. P3 enters the apartment, goes to the kitchen, and sits on another chair
4. P4 enters the apartment and goes to the kitchen
5. P4 goes in the bathroom
6. P4 comes back to the living room
7. P1 goes to the bathroom

## 5. Conclusions and Future Work

We have presented the E-care@home system, its installation at the Ängen research and innovation apartment, and a series of data sets collected using the system. The main contribution of this paper is the release of the data set that can be used by others. In addition, we have shown how a modular approach makes the E-care@home system useful as a whole or by allowing the usage of individual parts. This will provide a baseline for future developments as well as research projects and for students who need to collect data in realistic settings.

In E-care@home, we have created a permanent installation of a smart home sensor and reasoning system in the Ängen research and innovation apartment. We are currently heavily extending the set of sensors and sensor modules to facilitate the collection of richer data sets.

In future work, we intend to use this as a test bed for integrated smart home and AI technology for applications targeted at supporting people with dementia. As part of this initiative, we will extend the AI aspects of the E-care@home system to include data-driven methods and habit recognition. We also aim to fully integrate the configuration planner as a module that can be used to configure the E-care@home system based on its context and information goals. This will allow testing and evaluation based on feedback by various stakeholders and end users. It also sets the stage for developing smart home AI solutions capable of long-term autonomy.

## Figures and Tables

**Table 1:** The sensors used for the different scenarios: The two temperature sensors (id 1947 and 1965) are two different sensors located in the main body of the apartment and measure the same temperature.

Sensor id	Sensor Name	Type	Sending Frequency (Hz)
**Real Apartment Data Set**			
1846	bathroom/ambience/motion	INT	0.3
1847	kitchen/ambience/motion	INT	1
1851	livingroom/ambience/motion	INT	1
1852	livingroom/couch/pressure	INT	1
1941	kitchen/chair_1/pressure_1	INT	1
1942	kitchen/chair_1/pressure_2	INT	1
1947	$no_{l}ocation/node/temperature$	FLOAT	0.2
1948	kitchen/stove_led/luminosity	INT	0.2
1965	$no_{l}ocation/node/temperature$	FLOAT	0.2
1966	livingroom/tv_led/luminosity	INT	0.2
**Bedroom Data Set**			
3026	bedroom/bed_ambience_right/motion	INT	1
3031	bedroom/bed_ambience_left/motion	INT	1
3032	bedroom/bed/pressure	INT	1
3033	bedroom/ambience/motion	INT	0.3
3041	body/ankle/motion	INT	10
3042	body/wrist/motion	INT	10
3043	body/heart/rate	INT	10
**Person Counting Data Set**			
3206	livingroom/ambience/motion_1	INT	1
3209	livingroom/couch/pressure	INT	1
3243	kitchen/cabinet_4/door	INT	1
3270	kitchen/ambience/motion	INT	1
3271	kitchen/microwave/door	INT	1
3347	bathroom/ambience/motion	INT	1
3364	entrance/ambience/motion	INT	1
3457	kitchen/chair_1/pressure	INT	1
3476	kitchen/chair_2/pressure	INT	1

**Table 2:** List of activities labeled by the user living in the apartment, where location_id = 211 and name = ORU/Home1.

Annotation id	Name	Description
132	in,bathroom	user is in the bathroom
139	watchingtv,tv_led	user is watching TV
127	sitting,couch	user is sitting on the couch
136	cooking,stove_led	user is using the stove to cook
151	sitting,chair_1	user is sitting on chair_1
158	eating,chair_1	user is sitting on chair_1 to eat
179	resting	user is resting

**Table 3:** Wearable sensors used for the bedroom data set.

Sensor id	Name	Description
3041	body/ankle/motion	accelerometer (part of the shimmer) installed on the ankle of the user
3042	body/wrist/motion	accelerometer (part of the shimmer) installed on the wrist of the user
3043	body/heart/rate	(part of the shimmer) installed on the chest of the user

**Table 4:** List of activities labeled by the user living in the apartment, where location_id = 416 and name = ORU/Angen.

Annotation id	Name	Description
203	ds-lying-down-left	user is lying down on the left side of the bed
201	ds-getting-up-left	user is getting up from the left side of the bed
204	ds-lying-down-right	user is lying down on the right side of the bed
202	ds-getting-up-right	user is getting up from the right side of the bed
207	ds-sitting-on-bed-left	user is sitting on the left side of the bed
208	ds-sitting-on-bed-right	user is sitting on the right side of the bed
205	ds-sleeping	user is sleeping
206	ds-turning-in-bed	user is turning while (s)he is in the bed

**Table 5:** Annotations used for the ground truth about the number of occupants for the person counting data set.

Annotation id	Name
145	occupant,0
144	occupant,1
146	occupant,2
147	occupant,3
148	occupant,4

**Table 6:** Start and end times for the different instances of the three scenarios.

Scenario	Instance	Start Time	End Time	Data Set Label
01	1	19 September 2019 10:57:56	19 September 2019 11:02:07	ORU/Angen/PersonCounting/01/01
	2	19 September 2019 11:02:31	19 September 2019 11:05:56	ORU/Angen/PersonCounting/01/02
	3	19 September 2019 11:07:00	19 September 2019 11:10:07	ORU/Angen/PersonCounting/01/03
02	1	19 September 2019 10:24:27	19 September 2019 10:31:15	ORU/Angen/PersonCounting/02/01
	2	19 September 2019 10:32:29	19 September 2019 10:37:06	ORU/Angen/PersonCounting/02/02
	3	19 September 2019 10:38:12	19 September 2019 10:42:45	ORU/Angen/PersonCounting/02/03
03	1	19 September 2019 10:46:33	19 September 2019 10:49:04	ORU/Angen/PersonCounting/03/01
	2	19 September 2019 10:50:18	19 September 2019 10:52:51	ORU/Angen/PersonCounting/03/02
	3	19 September 2019 10:54:27	19 September 2019 10:56:48	ORU/Angen/PersonCounting/03/03
